# Proteinuria, ^**99m**^Tc-DTPA Scintigraphy, Creatinine-, Cystatin- and Combined-Based Equations in the Assessment of Chronic Kidney Disease

**DOI:** 10.1155/2014/430247

**Published:** 2014-02-11

**Authors:** Hernán Trimarchi, Alexis Muryan, Agostina Toscano, Diana Martino, Mariano Forrester, Vanesa Pomeranz, Fernando Lombi, Pablo Young, María Soledad Raña, Alejandra Karl, M. Alonso, Mariana Dicugno, Clara Fitzsimons

**Affiliations:** ^1^Servicios de Nefrología, Hospital Británico de Buenos Aires, Perdriel 74, 1280 Buenos Aires, Argentina; ^2^Laboratorio Central, Hospital Británico de Buenos Aires, Perdriel 74, 1280 Buenos Aires, Argentina; ^3^Servicios de Medicina Nuclear, Hospital Británico de Buenos Aires, Perdriel 74, 1280 Buenos Aires, Argentina; ^4^Servicios de Clínica Médica, Hospital Británico de Buenos Aires, Perdriel 74, 1280 Buenos Aires, Argentina

## Abstract

*Background*. Precise estimation of the glomerular filtration rate (GFR) and the identification of markers of progression are important. We compared creatinine, cystatin, and combined CKD-EPI equations with ^99m^Tc-DTPA scintigraphy to measure GFR and proteinuria as markers of progression. *Methods*. Cross-sectional, observational study including 300 subjects. CKD was classified by ^99m^Tc-DTPA scintigraphy. *Determinations*. Creatinine, 24-hour creatinine clearance, cystatin, Hoek formula, and creatinine, cystatin, and combined CKD-EPI equations. *Results*. In the global assessment, creatinine CKD-EPI and combined CKD-EPI equations yielded the highest correlations with ^99m^Tc-DTPA: **ρ** = 0.839, *P* < 0.0001 and **ρ** = 0.831, *P* < 0.0001. Intergroup analysis versus ^99m^Tc-DTPA: control G, creatinine clearance **ρ** = 0.414, *P* = 0.013; G3, combined CKD-EPI **ρ** = 0.5317, *P* < 0.0001; G4, Hoek **ρ** = 0.618, *P* < 0.0001, combined CKD-EPI **ρ** = 0.4638, *P* < 0.0001; and G5, creatinine clearance **ρ** = 0.5414, *P* < 0.0001, combined CKD-EPI **ρ** = 0.5288, *P* < 0.0001. In the global assessment, proteinuria displayed the highest significant correlations with cystatin (**ρ** = 0.5433, *P* < 0.0001) and cystatin-based equations (Hoek: *ρ* = −0.5309, *P* < 0.0001). When GFR < 60 mL/min: in stage 3, proteinuria-cystatin (**ρ** = 0.4341, *P* < 0.0001); proteinuria-Hoek (**ρ** = −0.4105, *P* < 0.0001); in stage 4, proteinuria-cystatin (**ρ** = 0.4877, *P* < 0.0001); proteinuria-Hoek (**ρ** = −0.4877, *P* = 0.0026). *Conclusions*. At every stage of GFR < 60 mL/min, cystatin-based equations displayed better correlations with ^99m^Tc-DTPA. Proteinuria and cystatin-based equations showed strong associations and high degrees of correlation.

## 1. Introduction

In clinical practice, it is critical to assess kidney function in a precise and accurate manner. Measurement of the glomerular filtration rate (GFR) is considered the best method that reflects kidney function, both in health and in disease [1]. The *Kidney Disease Outcomes Quality Initiative (K/DOQI) guidelines*, widely employed in clinical practice, stratify CKD into 5 stages according to the GFR estimated through the depuration of creatinine [2]. During the last decades, serum creatinine has been the most frequently employed marker to estimate GFR. The K/DOQI guidelines emphasize the necessity to assess GFR employing equations based on serum creatinine and not to rely on serum creatinine concentration alone [2]. The most commonly used creatinine-based formulae include Crockoft-Gault, adjusted to age, weight, and gender, and the *Modification of Diet in Renal Disease* (MDRD) and its variants, focused on estimating GFR [3]. Finally, *Chronic Kidney Disease Epidemiology* (CKD-EPI) equation, published in 2009 appears to be more exact than the previous ones in estimating GFR [1]. All these formulae lack the proper validation at the GFR at which they were applied because the creatinine methods were not standardized among the intervening centers, giving rise to differences in creatinine measurements [2, 4]. Finally, creatinine-based estimations of GFR present many drawbacks and depend on many variables, and the precision of these equations remains under intense debate [5, 6].

Cystatin C has been proposed as a new endogenous marker of GFR. This low molecular weight cysteine-protease inhibitor (13,300 Da) is produced at a constant rate by all the nucleated cells of the human body [7]. Cystatin C appears to protect connective tissue from intracellular enzymatic destruction and exerts antibacterial and antiviral actions [8]. Cystatin C is freely filtered through the glomerulus and is reabsorbed and metabolized but not secreted by the proximal tubule [8]. Consequently, serum cystatin C concentration depends almost exclusively on GFR [7]. Serum cystatin C concentration appears to be independent of muscular mass, gender, age, or nutritional status [7, 9], although recent studies have questioned these findings [10, 11]. Serum cystatin C levels may not be altered by inflammation, fever, or other agents [9]. Moreover, it appears to be a better marker of GFR in especial clinical conditions as hepatic cirrhosis and diabetes mellitus and in the elderly [12, 13]. Due to these properties, many have proposed cystatin C as a more precise marker of GFR than creatinine, particularly in subjects with mild damage of GFR [7, 14], but these studies are not only scant, but also contradictory and small in the number of patients included [9, 15–17]. However, some studies have also demonstrated that cystatin C appears to better identify CKD patients with a higher risk of cardiovascular complications at GFR < 60 mL/min estimated by creatinine CKD-EPI [18]. Despite the apparent theoretical advantages of cystatin C and the more polished equations, the debate continues and no equation has firmly been established to assess GFR at any stage [19]. Therefore, the necessity of newer equations is mainly due to the lack of precision to estimate GFR, particularly when the gold standard methods of GFR measurement vary from work to another [19]. Many equations based on creatinine and cystatin C have been developed. In this regard, we have recently published a prospective study in which kidney function was evaluated employing ^99m^Tc-DTPA as the gold standard of GFR and creatinine or cystatin C equations [6]. In that manuscript in which 300 subjects were included the main conclusions were that at GFR < 60 mL/min, CKD-EPI creatinine and cystatin C-based Hoek equations gave the best correlations with ^99m^Tc-DTPA. In controls and at early stages of CKD, creatinine-based equations correlated better with ^99m^Tc-DTPA, being creatinine clearance for controls and Cockroft-Gault equation for stages 1 and 2 the ones with the best degrees of agreement [6].

A developed creatinine-cystatin C (combined) CKD-EPI equation has been tested by some studies. In one cross-sectional work which included 1119 participants from 5 different studies, Inker et al. concluded that the combined creatinine-cystatin C CKD-EPI equation performed better than those based on either of these markers alone and may be useful as a confirmatory test for CKD [20].

In turn, CKD is an important and growing public health problem worldwide. It is estimated that approximately 500,000,000 people present some degree of kidney dysfunction [21–23]. One of the most important markers of CKD and its progression is proteinuria. Proteinuria is another predictor of increased cardiovascular risk in the general population [24]. Numerous studies have shown that treating proteinuria in patients with diabetic or nondiabetic CKD slows the progression of renal disease. It can also be stated that the greater the decrease in proteinuria, the greater the clinical benefit [25–27]. In addition to predicting kidney disease progression, proteinuria is a well-established risk marker for cardiovascular disease [28–31]. In CKD individuals, reduction in proteinuria confers a significant decrease in cardiovascular events. For example, the RENAAL study showed that albuminuria is the most important factor in predicting the cardiovascular risk in patients with type 2 diabetic nephropathy, and at 6 months for every 50% reduction in albuminuria, an 18% reduction in cardiovascular risk and a 27% reduction in heart failure were reported [32]. Finally, reduction of proteinuria by >30% within the first 6 to 12 months of treatment in patients with chronic kidney disease has also been shown to predict long-term renal and cardiovascular outcomes [24, 28, 33]. One question to be addressed is the role proteinuria plays—if any—in CKD stage 5 [34].

We decided to include the combined creatinine-cystatin C-based CKD-EPI equation and proteinuria measurements in the 300 patients previously studied and correlated them in terms of GFR at the different CKD stages. Finally, we also evaluated whether any correlation difference was encountered between proteinuria with either creatinine- or cystatin C-based equations at the different stages of CKD.

## 2. Methods

### 2.1. Study Design

Prospective, cross-sectional, observational study was undertaken between October 2009 and September 2010 and reassessed with respect to other GFR equations and included proteinuria as another variable. This reassessment was undertaken in March 2013 at the Hospital Británico de Buenos Aires, Argentina. Three hundred adult patients were included.

### 2.2. Regulatory Aspects

The study was approved by the Institutional Review Board (approval number 338). Cystatin C kits were donated by Gentian, Inc., Oslo, Norway. Local permissions by the Ministry of Health and ANMAT-INAME were obtained (form 788/0509, May 13, 2009).

### 2.3. Population

Three hundred Caucasian adult outpatients between 18 and 80 years were included. Gender: males, 174 (58%); females, 126 (42%). Chronic kidney disease and its stages were defined according to K/DOQI guidelines [1]: criterion number 1. Renal damage > 3 months, established by structural or functional damage, with or without decrease in GFR, shown by histological anomalies and renal damage markers, including those found in blood, urine, or images; or else, criterion number 2: GFR < 60 mL/min/1.73 m^2^> 3 months, with or without renal damage. In turn, National Kidney Foundation and K/DOQI guidelines divide CKD into 5 stages [1]; we also included a control group, which was defined as subjects without hypertension, diabetes mellitus, thyroid disease, one kidney, cancer, or previous episodes of renal disease, microhematuria or proteinuria, and with a normal renal sonogram.

### 2.4. Performed Studies

The following studies were performed: fasting serum creatinine and cystatin C, 24-hour creatinine clearance, 24-hour proteinuria, ^99m^Tc-DTPA scyntigraphy, and renal sonogram. Blood samples and gammagraphic studies were all done at the Hospital Británico facilities by the same professionals.

GFR was estimated by serum creatinine, 24-hour creatinine clearance, CKD-EPI creatinine [2], serum cystatin C, Hoek formula, CKD EPI cystatin C; and CKD EPI creatinine-cystatin C (combined) equations [22], and dynamic gammagraphy with ^99m^Tc-DTPA as the gold standard [35].

Creatinine was determined by dry chemistry sarcosine oxidase method with traceable calibration to mass spectrometry isotopic dilution using Vitros 5.1 FS autoanalyzer (*Johnson & Johnson, New Jersey, USA*). Total error of creatinine determination: 9.8% (Total error recommended: <10% according to NKDEP: *National Kidney Disease Education Program*—http://nkdep.nih.gov/). Method bias: 0.0056 (recommended method bias: <0.05). Normal levels: serum creatinine in males, 0.71–1.12 mg/dL; in females, 0.57–1.02 mg/dL. 

*Creatinine Based Calculations*

 Creatinine clearance: determined adjusted to age, weight, and height according to DuBois body surface area equation. The correct urine collection was tested by Walser equation.
 Twenty-four-hour urine creatinine clearance:
   GFR = urinary creatinine (mg/dL) (Serum creatinine (mg (dL))) × daily urinary output (1440) × DuBois body surface area (1.73 m^2^).
 CKD EPI:
   In males, if creatinine ≤ 0.9, GFR = 141 × (plasmatic creatinine (0.9))^−0.411^  × 0.993^age^.
   In males, if creatinine > 0.9, GFR = 141 × (plasmatic creatinine (0.9))^−1.209^ × 0.993^age^.
   In females, if creatinine ≤ 0.7, GFR = 144 × (plasmatic creatinine (0.7))^−0.329^  × 0.993^age^.
   In females, if creatinine > 0.7, GFR = 144 × (plasmatic creatinine (0.7))^−1.209^  × 0.993^age^.

 DuBois equation for body surface area calculation: BSA = 0.007184 × (weight kg)^0.425^  × (height cm)^0.725^.
 Walser formula:
   Males 28.2 − (0.172 × age).
   Females 21.9 − (0.115 × age).

*Cystatin C-Based Calculations.* Cystatin C was determined by immunoturbidimetry (Gentian Laboratory, Oslo, Norway), Vitros 5.1 FS (Johnson & Johnson, New Jersey, USA). Normal levels: 0.57–1.09 mg/L.

### 2.5. Equations

 Hoek: GFR = −4.32 + 80.35 × 1 (Cystatin C).
 CKD EPI:
   Female or male ≤0.8 133 × (cystatin C/0.8)^−0.499^ × 0.996^age^ [× 0.932 if female].
   Female or male >0.8 133 × (cystatin C/0.8)^−1.328^  × 0.996^age^ [× 0.932 if female].
 Combined CKD EPI:
 
*Female*
   If creatinine ≤ 0.7 or cystatin C ≤ 0.8
     130 × (creatinine/0.7) − 0.248 × (cystatin C/0.8) − 0.375 × 0.995^age^.
   If cystatin C > 0.8
     130 × (creatinine/0.7) − 0.248 × (cystatin C/0.8) − 0.711 × 0.995^age^.
 
*Female*
   
If creatinine > 0.7 or ≤0.8
     
* *130 × (creatinine/0.7) − 0.601 × (cystatin C/0.8) − 0.375 × 0.995^age^.
   If cystatin C > 0.8
     
* *130 × (creatinine/0.7) − 0.601 × (cystatin C/0.8) − 0.711 × 0.995^age^.
 
*Male*
   
* *If creatinine ≤ 0.9 or cystatin C ≤ 0.8
     135 × (creatinine/0.9) − 0.207 × (cystatin C/0.8) − 0.375 × 0.995^age^.
   
* *If cystatin C > 0.8
     135 × (creatinine/0.9) − 0.207 × (cystatin C/0.8) − 0.711 × 0.995^age^.
 
* *
*Male*
   
* *If creatinine > 0.9 or ≤0.8
     
* *135 × (creatinine/0.9) − 0.601 × (cystatin C/0.8) − 0.375 × 0.995^age^.
   
* *If cystatin C > 0.8
     
* *135 × (creatinine/0.9) − 0.601 × (cystatin C/0.8) − 0.711 × 0.995^age^.

^99m^Tc-DTPA gammagraphy was performed in all 300 subjects as the gold standard method to assess GFR and consequently stratify CKD [22].

### 2.6. Statistics

Results are expressed as the mean ± 2 standard deviations. Intergroup comparisons were analyzed with chi-square (*χ* ^2^), one-way ANOVA, and Mann-Whitney *U*  test for paired comparisons. Correlations between variables are expressed by Spearman coefficient. Results were considered significant if *P* ≤ 0.05. The Bland-Altman plots were used to compare the different estimates of the GFR.

## 3. Results

### 3.1. Global Analysis

Population analysis and GFR estimations estimated by other equations are depicted in Tables 1 and 2 and in [6]. Subjects were included in the different groups based on GFR measured by ^99m^Tc-DTPA scyntigraphy. Age and body mass index were different among groups; proteinuria, hypertension, diabetes, and primary glomerulopathies prevalence significantly increased as CKD worsened (Tables 1 and 2). In the global assessment, CKD-EPI creatinine and CKD-EPI combined equations yielded the highest correlations with ^99m^Tc-DTPA: *ρ* = 0.839, *P* < 0.0001 and *ρ* = 0.831, *P* < 0.0001 (Table 3). When correlations between the different equations were assessed, Hoek and cystatin CKD EPI yielded the highest result: *ρ* = 0.9851, *P* < 0.0001 (Table 4).

As to proteinuria, it displayed the highest significant correlations with serum cystatin C (*ρ* = 0.5433, *P* < 0.0001) and cystatin C-based equations (Hoek: *ρ* = −0.5309, *P* < 0.0001) and in stage 3 (*ρ* = 0.4341, *P* < 0.0001; Hoek *ρ* = −0.4105, *P* < 0.0001) and 4 (*ρ* = 0.4877, *P* < 0.0001; Hoek *ρ* = −0.4877, *P* = 0.0026) (Table 4).

### 3.2. Intergroup Analysis

Relevant differences emerged when groups were analyzed separately (Tables 5–10). Strongest significant correlations with ^99m^Tc-DTPA, in control G, creatinine clearance yielded the highest correlation: *ρ* = 0.414, *P* = 0.013. Moreover, all three CKD EPI equations showed significant degrees of correlations among themselves and with Hoek formula (Table 5). In G1 and 2, no significant correlations were obtained with respect to ^99m^Tc-DTPA, but the same pattern of high significance was observed among the different equations, being creatinine CKD EPI-combined CKD EPI the highest correlation obtained in stage 1 (*ρ* = 0.8522, *P* < 0.0001) and Hoek-cystatin C CKD EPI in stage 2 (*ρ* = 0.9496, *P* < 0.0001) (Tables 6 and 7). In G3, the highest correlation with ^99m^Tc-DTPA was obtained with combined CKD-EPI: *ρ* = 0.5317, *P* < 0.0001; and the highest degree of significance in the correlation analysis was obtained between Hoek and cystatin C CKD EPI: *ρ* = 0.9734, *P* < 0.0001 (Table 8). In G4, the highest correlation with ^99m^Tc-DTPA was obtained with Hoek equation (*ρ* = 0.618, *P* = 0.0001) and CKD-EPI combined (*ρ* = 0.5317, *P* < 0.0001). The most strong and significant correlation was obtained between Hoek formula and cystatin C CKD EPI (*ρ* = 0.9778, *P* < 0.0001) (Table 9). Finally, in stage 5 ^99m^Tc-DTPA-combined CKD-EPI formula (*ρ* = 0.5288, *P* < 0.0001) and ^99m^Tc-DTPA-creatinine clearance (*ρ* = 0.5414, *P* < 0.0001) comparisons gave the best results (Table 10).

**Figure 1:**
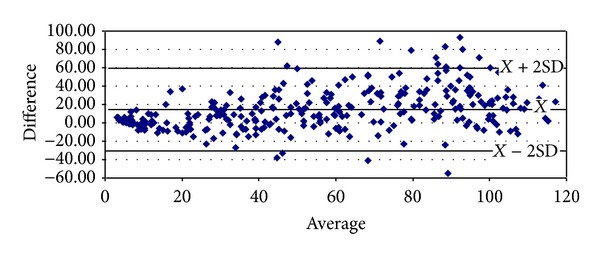
Bland-Altman plot between combined CKD-EPI and DTPA.

**Figure 2:**
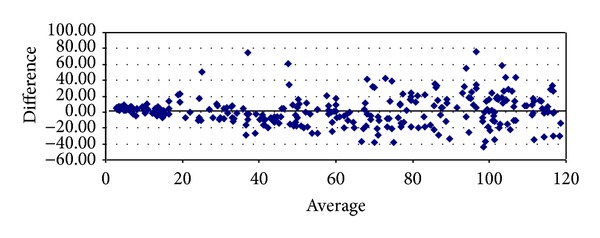
Bland-Altman plot between combined CKD-EPI and creatinine clearance.

**Figure 3:**
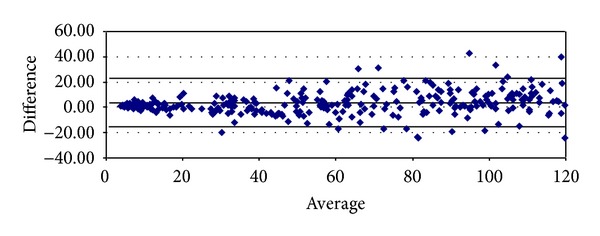
Bland-Altman plot between combined CKD-EPI and CKD-EPI creatinine.

**Figure 4:**
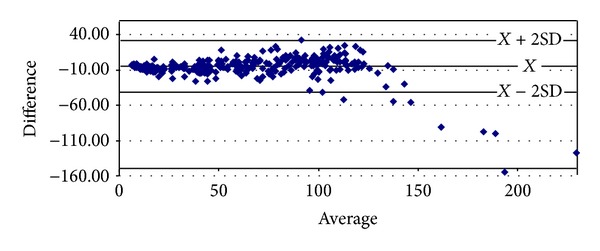
Bland-Altman plot between combined CKD-EPI and Hoek.

**Figure 5:**
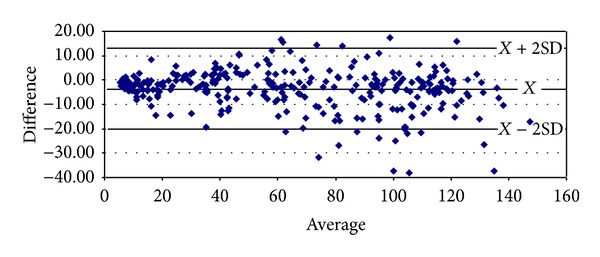
Bland-Altman plot between combined CKD-EPI and CKD-EPI cystatin.

**Figure 6:**
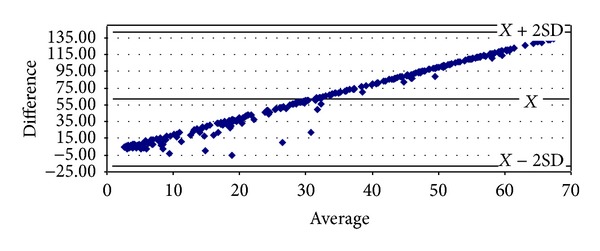
Bland-Altman plot between combined CKD-EPI and proteinuria.

With respect to proteinuria, it began to show significant correlations from stage 3 onwards (Tables 8–10). In stage 3, it presented strong degrees of correlation with all GFR methods of measurement, achieving the highest scores with cystatin C (*ρ* = 0.4341, *P* < 0.0001) and Hoek (*ρ* = −0.4140, *P* < 0.0001) (Table 8). The same pattern was ascertained in stage 4 with cystatin C (*ρ* = 0.4877, *P* = 0.0026) and Hoek (*ρ* = −0487, *P* = 0.0026) (Table 9). Finally, in G5 proteinuria was not found to correlate with any of the equations employed (Table 10). Bland-Altman plots were employed to illustrate the degree of agreement between combined CKD-EPI and the other different estimates of GFR (Figures 1, 2, 3, 4, and 5) and with respect to proteinuria (Figure 6).

## 4. Discussion

Briefly, in our previous recently published work, we have found that in the global 300-patient GFR evaluation, CKD-EPI and Hoek equations displayed the highest statistically significant correlations and the best lineal regressions with respect to ^99m^Tc-DTPA, and the different creatinine-based equations showed a high and significant correlation among themselves; the same phenomenon was reported with cystatin C-based formulas. Additionally, as GFR approached ≤60 mL/min, both serum creatinine and cystatin C concentrations, as their respective derived equations, converged to a better correlation among themselves, suggesting any equation valid to be employed. Finally, in stages 3 and 4, CKD-EPI and Hoek equations were the ones to best correlate with ^99m^Tc-DTPA [6].

However, combined creatinine-cystatin C equations had not been included in that work. After the publication by Trimarchi et al. and Shlipak et al. in which GFR was assessed by several methods in a multicenter study with 1119 subjects employing urinary clearance of iothalamate as the gold standard, including creatinine, cystatin C or combined CKD-EPI formulas, showing that the combined CKD-EPI equation was superior to equations based on either creatinine or cystatin C markers alone, we decided to consider cystatin C and the combined CKD-EPI equations to our previous available data [6, 20]. However, Shlipak et al. focused on the CKD-EPI equations alone.

In our present study, creatinine clearance was the best method to assess GFR in the control group (Table 5) [6]. In our previous work we had reported that for stages 1 and 2 of CKD, Cockroft-Gault was the one with the highest correlation and significance with respect to ^99m^Tc-DTPA [6]. We now have found that among subjects with GFR < 60 mL/min, at stage 3 combined CKD-EPI equations displayed the best correlation with ^99m^Tc-DTPA (Table 8). However, at stage 4 Hoek equation had a higher correlation with ^99m^Tc-DTPA and at stage 5 again combined CKD-EPI equation displayed the highest correlation with ^99m^Tc-DTPA (Tables 9 and 10). These findings may suggest that in chronic renal insufficiency, cystatin C may offer certain advantages over creatinine for GFR estimation when ^99m^Tc-DTPA is employed. We realize that this finding could change whether other gold standard methods to assess GFR are to be used. Noteworthy, the strong significant correlations found among all equations when compared among themselves at any stage of the disease suggest that they could be interchangeably employed to assess GFR, reinforced by the high degree of agreement (Tables 6–10 and Figures 1–5). Consequently, this finding may also suggest that creatinine can still be considered as a practical, non expensive molecule for GFR determination. Creatinine CKD-EPI appears to be better than the other creatinine-based equations [6]. However, according to our present results, when cystatin C is available, the combined CKD-EPI equation should be employed.

Besides, the main purpose of the development of newer equations should be focused on a higher precision of early detection of renal damage. Why is it important to focus on a correct GFR estimation at these early stages of CKD? It is important due to the large quantity of false negative CKD cases that are apparently being reported using the current methods [3, 14]. This is the spectrum of CKD with the greatest potential degree of recovery or preservation of renal function when appropriately diagnosed. We regrettably were unable to find a superiority of the combined CKD-EPI equation at stages 1 and 2 of CKD in our population (Tables 6 and 7).

When proteinuria was analyzed with respect to GFR ≤ 60 mL/min, it presented an inverse strong significant correlation with ^99m^Tc-DTPA, particularly at stages 3 and 4 (Tables 8 and 9). With respect to creatinine- and cystatin C-based equations, proteinuria presented better and significant positive correlations with cystatin C and inverse with all cystatin C-based equations (Hoek and cystatin CKD-EPI) and a lower—despite significant—degree of correlation with both creatinine and combined CKD-EPI formulas (Figure 6). Albeit we have previously studied the role proteinuria may play in patients on stage 5D, no firm conclusions can be drawn at the moment, as literature data is scant. In terms of proteinuria and CKD, it is well established that proteinuria is a marker of kidney disease progression and is estimated to augment as kidney function worsens [34, 36]. Moreover, proteinuria is a marker of cardiovascular risk, the main cause of mortality in CKD [34]. The relationship between proteinuria and cystatin C is interesting to analyze. Considering the fact that at these stages of CKD there exists a high correlation between proteinuria and ^99m^Tc-DTPA scyntigraphy, and higher correlations between proteinuria and cystatin C-related equations when compared to creatinine-based ones, a tempting hypothesis to explain this situation is that ^99m^Tc-DTPA and cystatin C themselves are only filtered by the glomerulus. In this situation, one should assume that in our population proteinuria is composed of albumin, which is exclusively filtered by the glomerulus as well. This phenomenon could also explain the lower correlations encountered between proteinuria and creatinine, as this molecule is excreted by the glomerulus and the proximal tubule. Noteworthy, it has recently been published that in the Asian population, an elevated serum cystatin C level could also be considered as an independent predictor of cardiovascular events in subjects with normal renal function, as it has been demonstrated for age and hypertension [37]. In this normal population, proteinuria has not been assessed. In addition, in elderly subjects with GFR > 60 mL/min that were studied, cystatin C has been shown to be a stronger predictor of the risk of death and cardiovascular events in elderly persons than creatinine is [20]. In a report in which other factors rather than GFR affected cystatin C levels, Stevens et al. found a strong association between proteinuria and higher cystatin C levels. The authors suggest that it could be due to a higher prevalence of diabetics in their population a finding that is also present in our study (Table 2) [6, 38]. In other studies, the stronger association between cystatin C with mortality and cardiovascular disease was ascribed to other variables, as older age and higher BMI [39–41]. In our study, higher cystatin C is associated with these two variables as CKD worsens (Table 1) and correlates with proteinuria (Tables 8–10), also prevalent in the elderly and the obese [6].

With regard to the limitations of the present study, the cohort was not matched according to gender or age, and the different BMI varied significantly among the stages (Table 1), which could have certainly influenced the results. Moreover, ^99m^Tc-DTPA scyntigraphy was used as the gold standard for GFR measurement, but it is not used routinely due to its cost, being time consuming and laborious [35]. Particularly, in CKD stages 1 and 2 and in the control group, microalbuminuria was not assessed; this consideration is relevant when early detection of CKD is pursued. Finally, the number of patients included and the ethnical features must be taken into account when conclusions or extrapolations are to be done.

Kidney function assessment with combined CKD-EPI appears to be superior to other equations when ^99m^Tc-D TPA scyntigraphy is employed at GFR < 60 mL/min but shows no advantages at earlier stages of kidney disease. Cystatin C is a more expensive determination, not applicable in many nephrology facilities, and with certain evidence of advantages compared with creatinine, and may be a better surrogate of CKD with respect to cardiovascular risk and proteinuria. However, cystatin C role in nephrology and as a useful tool to measure GFR in CKD has not been established yet, and more clinical data is needed. Finally, we believe that combined CKD-EPI equations require ample validation before being introduced for CKD staging.

## Figures and Tables

**Table 1:** General data of certain variables at different stages of CKD.

Confidence interval for the mean at 95%						
	Group	Media	Lower limit	Upper limit	Standard deviation	Interquartile amplitude
BMI	Control	23.95	22.690	25.217	3.68	2.63
	1	25.74	23.982	27.503	4.36	7.56
	2	25.95	24.764	27.133	4.26	5.47
	3	26.95	26.007	27.888	4.49	6.45
	4	29.29	27.170	31.418	6.37	6.51
	5	26.38	25.160	27.593	4.71	6.12

AGE	Control	48.63	44.25	53.006	12.74	16.00
	1	42.89	37.48	48.294	13.39	17.00
	2	45.23	41.41	49.051	13.72	18.75
	3	54.44	51.28	57.610	15.12	24.00
	4	63.22	58.47	67.963	14.24	14.50
	5	61.33	56.90	65.771	17.18	16.75

PROTEINURIA	Control	.049	.012	.087	.11	.00
	1	.37	.135	.598	.57	.41
	2	.31	.183	.426	.43	.40
	3	1.39	.708	2.075	3.26	1.14
	4	1.87	.808	1.560	.99	.71
	5	2.48	1.516	3.440	3.724	2.83

^ 99m^Tc-DTPA	Control	81.53	73.308	89.753	13.94	29.57
	1	95.26	88.605	101.944	15.80	15.77
	2	70.05	66.903	73.194	11.30	14.33
	3	45.59	43.624	47.556	9.39	14.27
	4	22.60	20.653	24.547	5.75	6.91
	5	11.18	8.989	13.364	8.40	9.79

Cystatin C	Control	.748	.717	.780	.092	.120
	1	.824	.724	.925	.249	.172
	2	.935	.846	1.024	.320	.364
	3	1.32	1.199	1.437	.569	.645
	4	1.98	1.532	2.035	.755	.850
	5	4.03	3.666	4.397	1.414	2.268

Abbreviations: CKD: chronic kidney disease; BMI: body mass index.

**Table 2:** Most frequent causes of CKD.

CKD etiology (*n* = patients)	Group								
	0	1	2	3	4	5	Total	*χ* ^2^	*P*
Hypertension	0	10	28	56	33	48	175	80.30	*0.0001 *
Diabetes	0	5	5	12	14	17	53	26.77	*0.0001 *
Glomerulonephritis	0	9	18	30	10	18	85	20.65	*0.024 *
PKD	0	2	3	11	2	9	27	8.54	*0.13 *

Abbreviations: CKD: chronic kidney disease; PKD: polycystic kidney disease.

**Table 3:** Glomerular filtration rate measured by all approaches globally and by stages of chronic kidney disease.

	DTPA	Creatinine	Creatinine clearance	Hoek	Creatinine CKD-EPI	Cystatin C CKD-EPI	Combined CKD-EPI	Cystatin C	Proteinuria
DTPA									
rho	1								
*P*									
Creatinine									
rho	−0.8113	1							
*P*	0								
Creatinine clearance									
rho	0.7847	−0.8158	1						
*P*	0	0							
Hoek									
rho	0.7975	−0.8749	0.8208	1					
*P*	0	0	0						
Creatinine CKD-EPI									
rho	0.8397	−0.9541	0.8365	0.9021	1				
*P*	0	0	0	0					
Cystatin C CKD-EPI									
rho	0.798	−0.8566	0.8172	0.9851	0.9105	1			
*P*	0	0	0	0	0				
Combined CKD-EPI									
rho	0.8311	−0.9231	0.845	0.9673	0.9728	0.978	1		
*P*	0	0	0	0	0	0			
Cystatin C									
rho	−0.791	0.876	−0.8182	−0.9928	−0.9011	−0.9918	−0.9707	1	
*P*	0	0	0	0	0	0	0		
Proteinuria									
rho	−0.4845	0.5273	−0.4928	−0.5309	−0.5086	−0.5241	−0.5291	0.5433	1
*P*	0	0	0	0	0	0	0	0	

**Table 4:** Global assessment correlations.

	DTPA	Creatinine	Creatinine clearance	Hoek	Creatinine CKD-EPI	Cystatin C CKD-EPI	Combined CKD-EPI	Cystatin C	Proteinuria
DTPA									
rho	1								
*P*									
Creatinine									
rho	−0.8113	1							
*P*	0								
Creatinine clearance									
rho	0.7847	−0.8158	1						
*P*	0	0							
Hoek									
rho	0.7975	−0.8749	0.8208	1					
*P*	0	0	0						
Creatinine CKD-EPI									
rho	0.8397	−0.9541	0.8365	0.9021	1				
*P*	0	0	0	0					
Cystatin C CKD-EPI									
rho	0.798	−0.8566	0.8172	0.9851	0.9105	1			
*P*	0	0	0	0	0				
Combined CKD-EPI									
rho	0.8311	−0.9231	0.845	0.9673	0.9728	0.978	1		
*P*	0	0	0	0	0	0			
Cystatin C									
rho	−0.791	0.876	−0.8182	−0.9928	−0.9011	−0.9918	−0.9707	1	
*P*	0	0	0	0	0	0	0		
Proteinuria									
rho	−0.4845	0.5273	−0.4928	−0.5309	−0.5086	−0.5241	−0.5291	0.5433	1
*P*	0	0	0	0	0	0	0	0	

**Table 5:** Control group correlations.

	DTPA	Creatinine	Creatinine clearance	Hoek	Creatinine CKD-EPI	Cystatin C CKD-EPI	Combined CKD-EPI	Cystatin C	Proteinuria
DTPA									
rho	1								
*P*									
Creatinine									
rho	−0.3813	1							
*P*	0.0238								
Creatinine clearance									
rho	0.4145	−0.4519	1						
*P*	0.0133	0.0064							
Hoek									
rho	0.0704	0.1287	0.0785	1					
*P*	0.6877	0.4611	0.6541						
Creatinine CKD-EPI									
rho	−0.0249	−0.1967	−0.0196	0.452	1				
*P*	0.8869	0.2575	0.911	0.0064					
Cystatin C CKD-EPI									
rho	−0.0378	0.3552	−0.0031	0.899	0.5299	1			
*P*	0.8292	0.0363	0.986	0	0.0011				
Combined CKD-EPI									
rho	0.0084	0.0307	0.0401	0.7974	0.8418	0.8664	1		
*P*	0.9618	0.861	0.8193	0	0	0			
Cystatin C									
rho	−0.0547	−0.13	−0.0872	−0.9985	−0.4582	−0.9032	−0.8072	1	
*P*	0.7551	0.4567	0.6184	0	0.0056	0	0		
Proteinuria									
rho	−0.0539	−0.079	−0.2187	0.2217	0.1269	0.0108	0.0239	−0.1991	1
*P*	0.7583	0.6521	0.2068	0.2005	0.4676	0.9508	0.8918	0.2514	

**Table 6:** Stage 1 correlations.

	DTPA	Creatinine	Creatinine clearance	Hoek	Creatinine CKD-EPI	Cystatin C CKD-EPI	Combined CKD-EPI	Cystatin C	Proteinuria
DTPA									
rho	1								
*P*									
Creatinine									
rho	−0.3645	1							
*P*	0.0799								
Creatinine clearance									
rho	−0.333	−0.2031	1						
*P*	0.1118	0.3411							
Hoek									
rho	0.0961	−0.3498	0.1409	1					
*P*	0.6551	0.0938	0.5114						
Creatinine CKD-EPI									
rho	0.3765	−0.7355	0.1748	0.3753	1				
*P*	0.0698	0	0.414	0.0707					
Cystatin C CKD-EPI									
rho	0.0722	−0.445	0.1148	0.5932	0.507	1			
*P*	0.7375	0.0293	0.5933	0.0022	0.0115				
Combined CKD-EPI									
rho	0.3183	−0.7199	0.0791	0.4966	0.8522	0.8087	1		
*P*	0.1296	0.0001	0.7132	0.0136	0	0			
Cystatin C									
rho	−0.0453	0.5042	−0.0571	−0.7028	−0.3969	−0.8926	−0.751	1	
*P*	0.8335	0.012	0.7911	0.0001	0.0548	0	0		
Proteinuria									
rho	0.2517	0.1	0.2521	−0.1154	0.1099	−0.0882	−0.0523	0.2501	1
*P*	0.2355	0.6422	0.2347	0.5912	0.6093	0.6821	0.8083	0.2384	

**Table 7:** Group 2 correlations.

	DTPA	Creatinine	Creatinine clearance	Hoek	Creatinine CKD-EPI	Cystatin C CKD-EPI	Combined CKD-EPI	Cystatin C	Proteinuria
DTPA									
rho	1								
*P*									
Creatinine									
rho	−0.1853	1							
*P*	0.184								
Creatinine clearance									
rho	0.2314	−0.4016	1						
*P*	0.0955	0.0029							
Hoek									
rho	0.1178	−0.6026	0.3886	1					
*P*	0.4008	0	0.004						
Creatinine CKD-EPI									
rho	0.2172	−0.8178	0.4104	0.737	1				
*P*	0.1182	0	0.0023	0					
Cystatin C CKD-EPI									
rho	0.1633	−0.51	0.3121	0.9496	0.7661	1			
*P*	0.2427	0.0001	0.0229	0	0				
Combined CKD-EPI									
rho	0.1309	−0.684	0.3815	0.8995	0.9182	0.9394	1		
*P*	0.3501	0	0.0048	0	0	0			
Cystatin C									
rho	−0.0603	0.5762	−0.3461	−0.9855	−0.7272	−0.9668	−0.9111	1	
*P*	0.6713	0	0.012	0	0	0	0		
Proteinuria									
rho	−0.1512	0.0199	−0.1293	−0.104	−0.0046	−0.1073	−0.06	0.145	1
*P*	0.2848	0.8885	0.3608	0.4631	0.9742	0.4489	0.6727	0.3049	

**Table 8:** Group 3 correlations.

	DTPA	Creatinine	Creatinine clearance	Hoek	Creatinine CKD-EPI	Cystatin C CKD-EPI	Combined CKD-EPI	Cystatin C	Proteinuria
DTPA									
rho	1								
*P*									
Creatinine									
rho	−0.4567	1							
*P*	0								
Creatinine clearance									
rho	0.4212	−0.6293	1						
*P*	0	0							
Hoek									
rho	0.4869	−0.6661	0.6265	1					
*P*	0	0	0						
Creatinine CKD-EPI									
rho	0.5033	−0.909	0.7025	0.6755	1				
*P*	0	0	0	0					
Cystatin C CKD-EPI									
rho	0.5073	−0.641	0.6376	0.9734	0.7092	1			
*P*	0	0	0	0	0				
Combined CKD-EPI									
rho	0.5317	−0.8181	0.7324	0.9094	0.8994	0.9369	1		
*P*	0	0	0	0	0	0			
Cystatin C									
rho	−0.5045	0.688	−0.6359	−0.9864	−0.7016	−0.9873	−0.9295	1	
*P*	0	0	0	0	0	0	0		
Proteinuria									
rho	−0.3584	0.3975	−0.3531	−0.4105	−0.3289	−0.3881	−0.3928	0.4341	1
*P*	0.0005	0.0001	0.0006	0.0001	0.0015	0.0002	0.0001	0	

**Table 9:** Group 4 correlations.

	DTPA	Creatinine	Creatinine clearance	Hoek	Creatinine CKD-EPI	Cystatin C CKD-EPI	Combined CKD-EPI	Cystatin C	Proteinuria
DTPA									
rho	1								
*P*									
Creatinine									
rho	−0.4418	1							
*P*	0.001								
Creatinine clearance									
rho	0.5249	−0.6193	1						
*P*	0.001	0.0001							
Hoek									
rho	0.618	−0.8843	0.6417	1					
*P*	0.0001	0	0						
Creatinine CKD-EPI									
rho	0.463	−0.9433	0.6921	0.8519	1				
*P*	0.02	0	0	0					
Cystatin C CKD-EPI									
rho	0.4525	−0.8306	0.6759	0.9778	0.8486	1			
*P*	0.003	0	0	0	0				
Combined CKD-EPI									
rho	0.5638	−0.9105	0.7076	0.9356	0.956	0.9477	1		
*P*	0.001	0	0	0	0	0			
Cystatin C									
rho	−0.4998	0.8756	−0.6531	−0.9963	−0.8499	−0.9849	−0.939	1	
*P*	0.001	0	0	0	0	0	0		
Proteinuria									
rho	−0.4995	0.4186	−0.3834	−0.487	−0.3306	−0.4612	−0.39	0.4877	1
*P*	0.0003	0.0111	0.0001	0.0026	0.0489	0.0046	0.0187	0.0026	

**Table 10:** Group 5 correlations.

	DTPA	Creatinine	Creatinine clearance	Hoek	Creatinine CKD-EPI	Cystatin C CKD-EPI	Combined CKD-EPI	Cystatin C	Proteinuria
DTPA									
rho	1								
*P*									
Creatinine									
rho	−0.4491	1							
*P*	0.0004								
Creatinine clearance									
rho	0.5414	−0.7461	1						
*P*	0	0							
Hoek									
rho	0.4818	−0.5612	0.6473	1					
*P*	0.0001	0	0						
Creatinine CKD-EPI									
rho	0.4806	−0.9656	0.7601	0.5904	1				
*P*	0.0001	0	0	0					
Cystatin C CKD-EPI									
rho	0.4683	−0.4994	0.6202	0.9856	0.56	1			
*P*	0.0002	0.0001	0	0	0				
Combined CKD-EPI									
rho	0.5288	−0.8284	0.7759	0.8873	0.8815	0.8755	1		
*P*	0	0	0	0	0	0			
Cystatin C									
rho	−0.4707	0.5456	−0.6325	−0.9974	−0.5752	−0.9886	−0.8802	1	
*P*	0.0002	0	0	0	0	0	0		
Proteinuria									
rho	0.2921	0.0209	0.2756	0.2272	0.1053	0.2564	0.2029	0.2269	1
*P*	0.0248	0.875	0.0346	0.0835	0.4273	0.05	0.1233	0.0839	
